# Sex Difference in Lower-limb Electromyography and Kinematics when Using Resistance Bands during a Barbell Back Squat

**DOI:** 10.5114/jhk/159585

**Published:** 2023-01-20

**Authors:** Shahab Alizadeh, Leah Vardy, Garrick N. Forman, Davis A. Forman, Michael W. R. Holmes, Duane C. Button

**Affiliations:** 1 School of Human Kinetics and Recreation, Memorial University of Newfoundland, St. John’s, NL, Canada.; 2 Department of Kinesiology, Brock University, St. Catharines, ON, Canada.; 3 Department of Kinesiology, Trent University, Durham, ON, Canada.; 4 Faculty of Medicine, Memorial University of Newfoundland, St. John’s, NL, Canada.

**Keywords:** dynamic knee valgus, medial knee displacement, knee abduction, resistance band

## Abstract

The aim of this study was to compare the muscle activity of the gluteus medius (GMe), gluteus maximus (GMa), biceps femoris (BF), vastus lateralis (VL), vastus medialis (VM) and erector spinae (ES) as well as medial knee displacement (MKD) while using varying stiffness resistance bands (red: 1.68 kg; black: 3.31 kg; gold: 6.44 kg) during a barbell back squat (BBS) among males and females. A total of 23 (females: 11) resistance trained people were recruited for this study. Muscle activity was measured using electromyography, and motion capture cameras tracked lower-limb kinematics and MKD. Three resistance bands were placed at the distal end of the femur while performing a BBS at their 85% repetition maximum (RM). Parametric and non-parametric statistical analyses were conducted with the alpha level of 0.05. The gold resistance band resulted in a smaller knee-width-index value (i.e., greater MKD) compared to other bands (p < 0.01). Males exhibited less MKD compared to females during the BBS for each resistance band (p = 0.04). Males produced greater VL activity when using the black and gold resistance bands during the BBS (p = 0.03). When using a gold resistance band, the GMe muscle activation was higher compared to other resistance bands (p < 0.01). VM muscle activity was reduced when using a gold resistance band compared to no band condition (p < 0.01). BF (p = 0.39) and ES (p = 0.88) muscle activity did not change when using different resistance bands. As a result, females may be at a biomechanical disadvantage when using resistance bands compared to males while performing the BBS hindering them from optimal performance.

## Introduction

Back squats are a highly effective exercise to enhance force generation, increase total body mass, reduce the risk of injuries and improve core stability (Young, 2006). Squatting is also a valuable rehabilitation exercise and a screening tool to evaluate functional movement competency (Escamilla, 2001). In order to execute different variations of the squat movement (Monajati et al, 2019), both with and without various utilities (Joseph et al., 2020, Foley et al., 2017), coordination of many muscles and joints is required. During the squat it is important for the individual to consider knee alignment (Escamilla, 2001; Schoenfeld, 2010). When squatting many individuals experience medial knee displacement (MKD); a motion in which the knee deviates from its “natural” alignment toward the midline of the body (Padua and Hirth, 2007). One method to quantify MKD is by calculating the knee-width index (KWI) which can be determined as the ratio of distance between the right and the left distal thigh (lateral epicondyles) and the shank (lateral malleoli) (Foley et al., 2017; Gooyers et al., 2012). MKD has been suggested to be a risk factor for lower extremity injuries such as anterior cruciate ligament (ACL) tear and patellofemoral pain syndrome (Hewett et al., 2005; Myer et al., 2010). MKD can also alter muscle recruitment patterns and activity, as indicated by electromyography (EMG). For example, there is a concomitant decrease and increase in gluteal and adductor EMG, respectively, during squat movements with MKD (DeJong et al., 2019; Foley et al., 2017). A neuromechanical mechanism to potentially help limit the amount of MKD during the squat would be to abduct the thighs which could alter gluteal and abductor muscle activity. One particular muscle of interest when examining MKD during the squat is the gluteus medius (GMe). The GMe contributes to thigh abduction and rotation, thus, targeting the GMe during a squat may lead to reduced MKD.

One way to reduce MKD during a squat is to place a resistance band around the distal thighs. Resistance bands provide a force vector directed medially about the knee and to counteract MKD, GMe muscle activation is a desired strategy. Resistance bands are thought to act as a proprioceptive aid to modulate knee alignment during the squat. However, research to support the use of a resistance band as a proprioceptive aid for MKD during the squat is contradictory. Resistance bands either increase (Forman et al., 2021; Gooyers et al., 2012; Reece et al., 2020) or do not affect the amount of MKD (Foley et al., 2017). Furthermore, an increase (Foley et al., 2017; Spracklin et al., 2017) and no change (Forman et al., 2021; Reece et al., 2020) in GMe muscle activity when using a resistance band during a squat has been reported. The contradictory results between studies may be attributed to different resistances applied to the knees (due to the use of varying resistance bands), the load being squatted, the experience level and other factors.

Two studies that used light resistance bands, when compared to no band, reported no change in MKD during a squat (Gooyers et al., 2012; Reece et al., 2020). On the contrary, one study reported an increase in MKD in the participant’s right knee when using a light resistance band compared to no band condition during a squat (Reece et al., 2020). For a medium resistance band, one study showed no effect on MKD during weighted barbell back squats (Foley et al., 2017), while another study reported an increase in MKD during a bodyweight squat (Gooyers et al., 2012). Results from strong resistance bands are similar, in that they increase the amount of MKD during a squat (Forman et al., 2021; Reece et al., 2020), however, it is worth noting that the method for which studies determined MKD was different from one another. Only one study compared the effects of varied stiffness resistance bands on MKD, and a strong resistance band increased MKD during a squat compared to a light resistance band (Reece et al., 2020). Additionally, only two studies recruited females as part of their experiment (Gooyers et al., 2012; Reece et al., 2020), with one study reporting no difference in MKD between males and females (Reece et al., 2020). It is known that females have a greater Q-angle than males. The Q-angle is measured by the intersection of lines drawn from the anterior superior iliac spine to the center of the patella and from the center of the patella to the tibial tubercle (Conley et al., 2007; Herrington and Nester, 2004; Livingston, 1998). Despite the difference in MKD and Q-angle calculation, an increased Q-angle seems to predispose the knee to greater MKD during dynamic activities, such as a squat, causing deviation from a neutral knee alignment (Carson and Ford, 2011; Ferber et al., 2003; Russell et al., 2006). Although it seems that different resistance bands alter knee alignment during the squat, collectively no study has compared the effects of varied resistance bands (i.e., medium to strong) on MKD during a squat nor between sexes.

Similar to MKD, different resistance bands have resulted in equivocal reports for GMe muscle activity while squatting. Two studies showed that using light and extra strong resistance bands did not alter GMe activation during a squat (Forman et al., 2021; Reece et al., 2020), while another study reported a decrease in GMe activity when using a medium resistance band during a squat (Foley et al., 2017). Spracklin et al. (2017) on the other hand, reported an increase in GMe activity when utilizing strong resistance bands during the squat. Additionally, squatting without the use of resistance bands showed minor differences in biceps femoris (BF) activity between sexes (Mehls et al., 2020), but no differences in muscle activity were found between sexes when using resistance bands (Reece et al., 2020). While it seems that different resistance bands may alter GMe activity differently, no study has collectively examined the effect of different band resistance on GMe during a squat between sexes.

Thus, the aim of this study was to compare frontal plane knee kinematics and muscle activity in the dominant vastus medialis (VM), vastus lateralis (VL), BF, GMa, GMe and erector spinae (ES) when using different band resistances during a barbell back squat (BBS) in males and females. We hypothesised that with an increase in resistance band stiffness, the value of KWI would decrease and concomitantly increase the amount of GMe EMG activation. We also hypothesised that females would exhibit a smaller KWI compared to males when using different resistance bands during a squat.

## Methods

### Participants

Using G-Power (GPower 3.1, Dusseldorf, Germany) a sample size of 16 was recommended for a statistical power of 0.8 at an alpha level of 0.05. Twenty-three (12 males and 11 females, age: 22.6 ± 2.6 years, body mass: 74.4 ± 13.8 kg, body height: 172.9 ± 8.7 cm) resistance-trained individuals participated in the study. Resistance-trained individuals were defined as individuals with regular engagement in resistance training (3 or more times per week) for at least one year and familiarity with the barbell back squat. Participants also had no history of injury in the past year. After verbally explaining the procedure, participants signed a consent form. Additionally, participants completed the Physical Activity Readiness Questionnaire (PAR-Q+) in order to assess their exercise readiness. This study was in accordance with the Tri-Council guidelines in Canada and approved by the Interdisciplinary Committee of Ethics in Human Research of the Memorial University of Newfoundland (ICEHR #20192643-HK) as well as the Research Ethics Board at the Brock University (REB # 18-265).

### Measures

#### Five Repetition Maximum (5RM)

Strength was defined as the five-repetition maximum (5RM) squat relative to body mass (squat weight/body mass) for each participant. The total mass squatted equaled the mass of the barbell combined with the mass of the plates.

#### Electromyography (EMG)

Skin preparation for all electrodes consisted of hair removal with a disposable razor, skin abrasion, and cleansing with an isopropyl alcohol swab. To ensure consistent electrode placement, the same researcher placed the AgAgCl disposable electrodes (MediTrace 130, Kendall, Mansfield, MA, USA) over each muscle belly and in-line with muscle fiber orientation, according to previous work (Hermens et al., 2000). A ground electrode was placed on the fibular head. Muscle activity was recorded from the participant’s dominant leg vastus medialis (VM), vastus lateralis (VL), biceps femoris (BF), gluteus maximus (GMa), gluteus medius (GMe) and erector spinae (ES) using an 8-channel Bortec EMG system. EMG signals were differentially amplified and band pass filtered (10–1000 Hz; CMRR > 115 dB at 60 Hz; input impedance 10 G Ω; AMT-8, Bortec Biomedical Ltd., Calgary, AB, Canada) and sampled at 2160 Hz. Muscles were located using SENIAM guidelines.

#### Kinematics

**Figure 1A:**
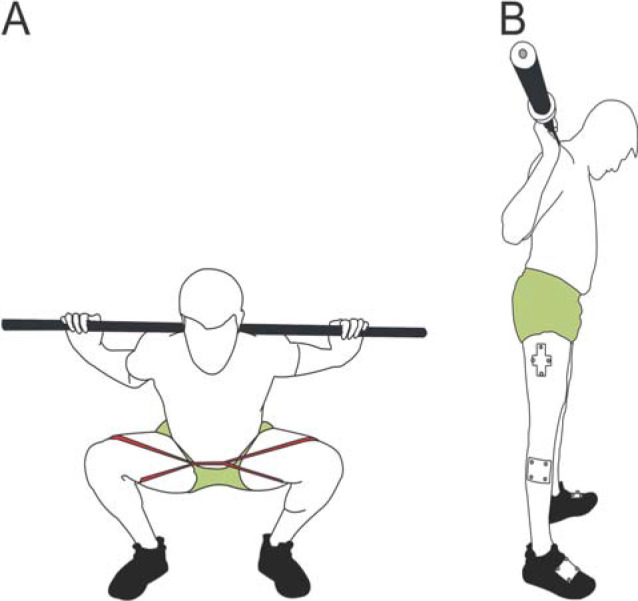
Loop resistance band placement while squatting, **1B**. Reflective marker placement on the thigh, shank, and foot.

Three-dimensional kinematics of the lower extremity were measured using a 10-camera Vicon motion capture system (VICON, Oxford, UK). Custom-designed, rigid bodies, consisting of four spherical reflective markers were attached to the left and the right foot, shank and thigh (Figure 1B) as well as the pelvis and thorax, approximately at mid-segmental regions. Static calibration markers, placed on the medial and lateral foot, malleoli, epicondyles, and greater trochanters, as well as the left and right posterior superior iliac spine and the acromion were removed during experimental collections assuming a fixed spatial relationship between anatomical landmarks and rigid bodies. Kinematics were sampled at 240 Hz and synchronized with the EMG data. A global coordinate system was calculated prior to the start of every session with X representing medial/lateral, Y representing anterior/posterior, and Z representing superior/inferior directions. Calibration involved participants standing in the anatomical position to generate a relationship between the rigid bodies and individual markers to allow digital recreation of segments and local coordinate systems in post processing.

#### TheraBand CLX

Three different resistance TheraBand CLX Consecutive Loops (The Hygenic Corporation, OH, USA) were used in addition to a no-resistance band condition. The TheraBand red, black, and gold bands, which provided 1.68 kg, 3.31 kg and 6.44 kg of resistance, respectively, at 100% elongation were used for all participants in randomized order. The bands were replaced after every third participant to ensure consistency between subjects. The resistance bands were placed around the distal quadriceps, just proximal to the patella so there was no interference with knee motion (Figure 1A).

### Design and Procedures

Participants were required to visit the laboratory on two separate occasions. The first experimental session was used to determine the participants’ 5 repetition maximum (RM), which is approximately 85% of 1RM (Brzycki, 1989). All participants completed a self-selected warm-up routine. Once the participant was sufficiently warmed up, their 5RM was determined by following ACSM guidelines (Swain and Brawner, 2012). No EMG or kinematics were collected in this session. Only the weight determined was used in session two. To determine 5RM, participants started squatting from 20.5 kg (weight of the barbell) without the resistance band. Weights were added in 5-10 kg increments in accordance with the ACSM warm-up guidelines until they reached their estimated 5RM (Swain and Brawner, 2012). Since participants were experienced athletes, they self-selected their own foot and hand-grip position during the squats. Once their foot placement was chosen, tape was placed on the ground to ensure that the position was consistent throughout the entirety of the session.

At the start of experimental session two, participants completed a warm-up routine consisting of a 5-min self-selected pace run on a motorised treadmill, followed by dynamic stretching of the lower extremity muscles and then several bodyweight squats. Similar to experimental session one, the warm-up was not standardized as participants were all resistance trained and experienced athletes. Participants used the same foot placement as in experimental session one. Then, reflective markers were attached with double sided tape. Next, electrodes were placed on the VM, VL, BF, GMa, GMe, and ES muscles. Participants then completed maximal isometric voluntary contractions (MVC) for each muscle. Then, participants began the BBS protocol with weight of the bar and no band. The weight on the barbell was then increased to the 5RM weight calculated from session one. Participants completed one set of three repetitions of the BBS for each of the conditions (no resistance band, the red, black, and gold TheraBand CLX) in randomized order for a total of four sets. After each set of 3 repetitions, a 5-min rest interval was assigned to prevent fatigue. Participants were instructed to perform the BBS at a controlled tempo of 2-0-2-1 (2 s eccentric descent, 0 hold at the bottom, 2 s concentric rise, 1 s hold at the top) at a metronome cadence of 50 bpm. Instructions were given to each participant prior to the addition of the band to maintain tension on the band throughout the entire repetition to prevent medial collapse of the knees.

### Data Analysis

EMG data were processed using full wave rectification and digitally filtered using a Butterworth low-pass filter (3 Hz cut-off, dual pass, 2^nd^ order). Muscle activity from all squat trials was normalized to their respective muscle peak EMG activity collected during the muscle specific MVCs using MATLAB (MathWorks Inc., Natick, MA, USA). The average EMG activity for each muscle was calculated separately from both ascending and descending phases of the squat.

Kinematic markers were manually labelled in Vicon Nexus 2.5 (VICON, Oxford, UK) and exported for further analysis using Visual3D (C-Motion, Germantown, MD, USA). Kinematic signal processing consisted of data filtering, local joint coordinate system construction, and calculation of the joint rotation sequence. Segment length and orthogonal coordinate systems were constructed using ISB standards (Wu et al., 2002). Kinematics were digitally filtered using a low-pass Butterworth filter with a cut-off frequency of 6 Hz (Forman et al., 2021). The maximum and minimum knee joint angles were used to examine the start of the descending and ascending phases for each repetition. The kinematic data were time normalized from 0-100% for each repetition of the squat. The KWI was calculated as a ratio of the distance between the right and left lateral femoral epicondyles to the right and left lateral malleoli (Foley et al., 2017).

### Statistical Analysis

The Shapiro-Wilk test was used to assess the normality of the data. The normality test revealed that EMG activity of the GMa, GMe, BF, ES, participant’s strength (5RM), and the KWI were normally distributed (*p* > 0.05). The EMG activity for the VL and VM, and the maximum knee flexion angle were not normally distributed in some of the conditions (*p* > 0.05), therefore nonparametric tests were used to compare these values. Multiple three-way factorial repeated measures ANOVAs dependent variables: EMG and KWI for each condition, independent variables: sex, phase and band) were used for statistical analysis. If the assumption of sphericity was violated based on the Mauchly's test, the Greenhouse–Geisser correction was used. An independent *t*-test was used to compare the difference between male and female strength. For data that were not normally distributed the Friedman test was used to compare the results. If a significant effect was observed, Bonferroni or Wilcoxon signed-rank post-hoc tests were used for parametric and nonparametric data, respectively, to identify the difference. Pearson’s correlation was used for parametric data, while Spearman’s correlation was used for non-parametric data. Alpha levels were set to 0.05 for all statistical tests. Partial eta-squared (pη2) measures indicating the magnitude of changes associated with significant main effects were provided and reported as small (< 0.01), medium (≥ 0.06) or large (≥ 0.14) (Cohen, 1992).

## Results

### EMG

#### Parametric Data

**Figure 2:**
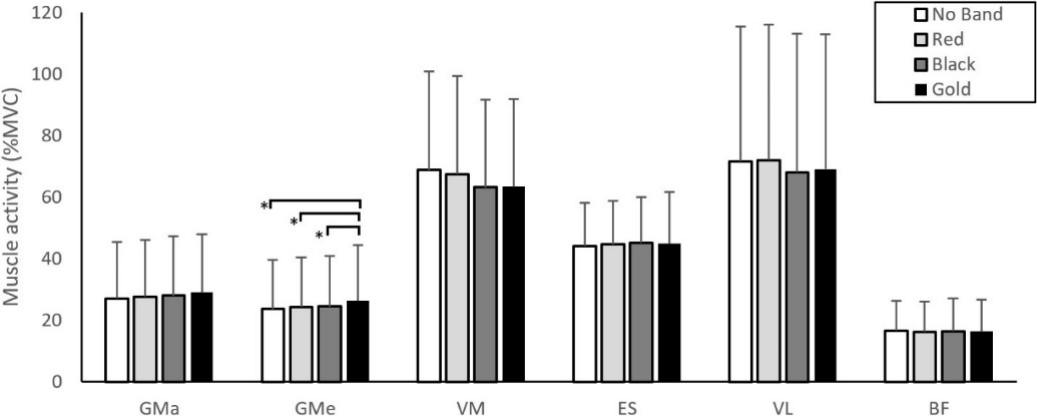
Gluteus Maximus (GMa), Gluteus Medius (GMe), Vastus Medialis (VM), Erector Spinae (ES), Vastus Lateralis (VL), and Biceps Femoris (BF) muscle activity for each resistance band condition (No Band, Red, Black, Gold). * p < 0.05

**Figure 3:**
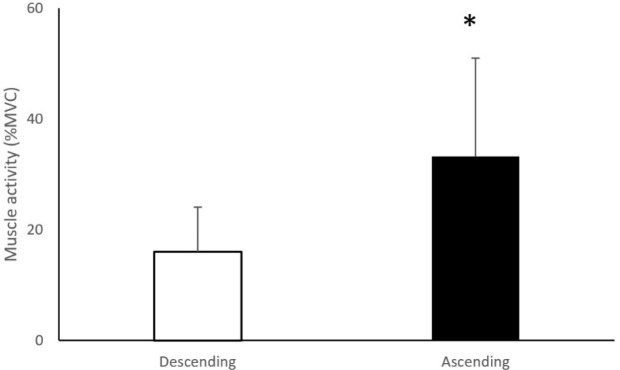
Gluteus Medius (GMe) muscle activity during the descending and the ascending phase of the barbell back squat. * *p* < 0.01

A summary of the EMG statistical findings is presented in Table 1. There was a significant main effect for the band on GMe muscle activity (F (1.75,70.05) = 10.17, *p* < 0.01, pη^2^ = 0.20) with the gold band showing significantly higher GMe muscle activity compared to all other conditions (no band: ↓11.4%, *p* < 0.01; red band: ↓7.7%, *p* < 0.01; black band: ↓6.4%, *p* < 0.01; Figure 2). No significant band × sex (*p* = 0.24), band × phase (*p* = 0.19), nor band × sex × phase (*p* = 0.43) interactions were found on GMe muscle activity. There was no significant effect for the band (*p* = 0.06) nor interaction effect (band × sex, *p* = 0.78; band × phase, *p* = 0.83; band × sex × phase, *p* = 0.59) on GMa muscle activity. There was no significant effect for the band (*p* = 0.88) nor interaction effect (band × sex, *p* = 0.11; band × phase, *p* = 0.62; band × sex × phase, *p* = 0.91) on ES muscle activity. There was no significant effect for the band, sex, and squat phase nor interaction effect on BF muscle activity (*p* = 0.06). There was a significant effect for the position on GMe muscle activation (F (1,20) = 52.32, *p* < 0.001, pη^2^ = 0.72; Figure 3) with GMe muscle activity being higher in the ascending phase compared to the descending phase.

**Table 1:** Statistically significant values for different muscle activity during the BBS.

Muscle	Band	Band × Sex	Band × Phase	Band × Sex × Phase
Gluteus Medius	*p* < 0.001*	*p* = 0.24	*p* = 0.19	*p* = 0.43
Gluteus Maximus	*p*= 0.06	*p*= 0.78	*p*= 0.83	*p*= 0.59
Erector Spinae	*p*= 0.89	*p*= 0.11	*p*= 0.62	*p*= 0.91
Biceps Femoris	*p*= 0.39	*p*= 0.29	*p*= 0.34	*p*= 0.71
Vastus Medialis	*p*< 0.001*	*p*= 0.16	*p*< 0.001*	*p*< 0.001*
Vastus Lateralis	*p*= 0.04*	*p*< 0.01*	*p*< 0.001*	*p*= 0.78

* : statistically significant

#### Non-Parametric Data

**Figure 4:**
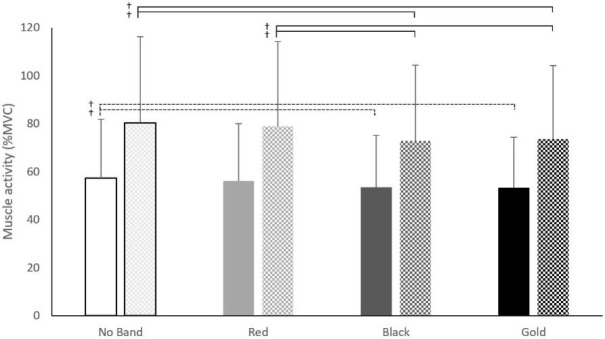
Vastus Medialis muscle activity during the ascending (checkered) and the descending (solid colour) phase of a barbell back squat among band conditions (No Band, Red, Black, Gold). †: *p <* 0.05

The nonparametric results revealed a main effect for the band on VM (ꭓ^2^(3) = 20.02, *p* < 0.01). The post-hoc Wilcoxon Signed Ranks test showed that the black band had a lower VM EMG activity compared to no band (↓8%, Z = 3.36, *p* < 0.01), red band (↓8%, Z = 2.52, *p* = 0.01) and gold band (↓4%, Z = 3.04, *p* < 0.01) condition. Additionally, the gold band produced lower EMG activity in the VM muscle compared to no band condition (↓5%, Z = 2.52, *p* = 0.01). A significant interaction effect for band × phase on VM muscle activity was shown (ꭓ^2^(7) =101.75, *p*<0.01). The post-hoc test showed that the black band (↓7%, Z = 2.26, *p* = 0.02) and the gold band (↓7%, Z = 2.45, *p* = 0.01) had a reduction in their VM muscle activity compared to no band condition during the descending phase (Figure 4). During the ascending phase both black (↓9%, Z = 3.13, *p* < 0.01) and gold (↓8%, Z = 2.32, *p* = 0.02) bands showed a reduction in the VM muscle activity compared to no band condition. Additionally, both black (↓7%, Z = 2.06, *p* = 0.04) and gold bands (↓7%, Z = 1.96, *p* = 0.04) showed lower VM muscle activity compared to the red band during the ascending phase. There was no band × sex interaction for VM ꭓ^2^(7) = 10.5, *p* = 0.16. A significant interaction effect was found for band × sex × phase (ꭓ^2^(15) = 88.25, *p* < 0.01). During the ascending phase of the squat males produced higher VM EMG values when using the gold band compared to females when using the red (↑6%, Z = 2.29, *p* = 0.03), black (↑13%, Z = 2.29, *p* = 0.02), and gold band (↑10%, Z = 2.29, *p* = 0.02).

**Figure 5:**
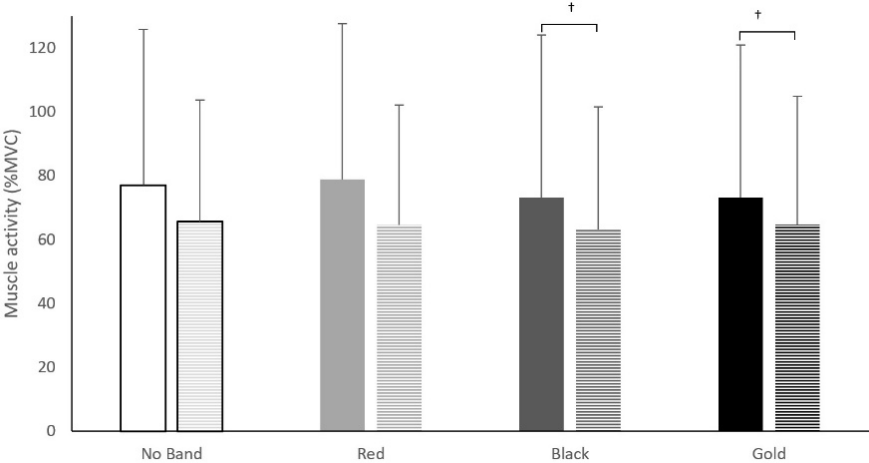
Vastus Lateralis (VL) muscle activity activity between males (solid colour) and females (stripes) for each band condition (No Band, Red, Black, Gold). †: *p* < 0.05

There was a main effect for the band (ꭓ^2^(3) = 8.13, *p* = 0.04) on the VL EMG activity. The post-hoc test showed a reduction in VL EMG activity when using the black band compared to no band (↓5%, Z = 2.32, *p* = 0.02) and red band (↓5%, Z = 2.22, *p* = 0.03). An interaction effect was found for band × phase (ꭓ^2^(7) = 99.64, *p* < 0.01). The post-hoc test showed an increase in the VL muscle activation when using the gold band compared to the black band during the descending phase (↑5%, Z = 2.14, *p* = 0.03). There was a significant interaction effect for band × sex for VL (ꭓ^2^(7) = 21.8, *p* < 0.01). The post-hoc test showed that the VL in males had a higher (↑10%) muscle activity when using the black band compared to females (Z = 1.99, *p* = 0.04, Figure 5). It was also found that males produced greater (↑8%) VL muscle activation compared to females when using the gold band (Z = 2.29, *p* = 0.02, Figure 5). No interaction effect was found for band × sex × phase (*p* = 0.78).

When not controlled for sex and the squat phase, no correlation was identified between the KWI and muscle activity (*p* = 0.06). However, when controlled for sex and the phase, females showed a negative correlation between the KWI and BF muscle activation while descending (r = -0.72, *p* = 0.01) when using the red band. During the ascending phase, females showed a positive correlation between the amount of the KWI and VM (r = 0.73, *p* = 0.01) and VL (r = 0.66, *p* = 0.03) muscle activity when using the black band. No other correlation was found.

### Kinematics

**Figure 6:**
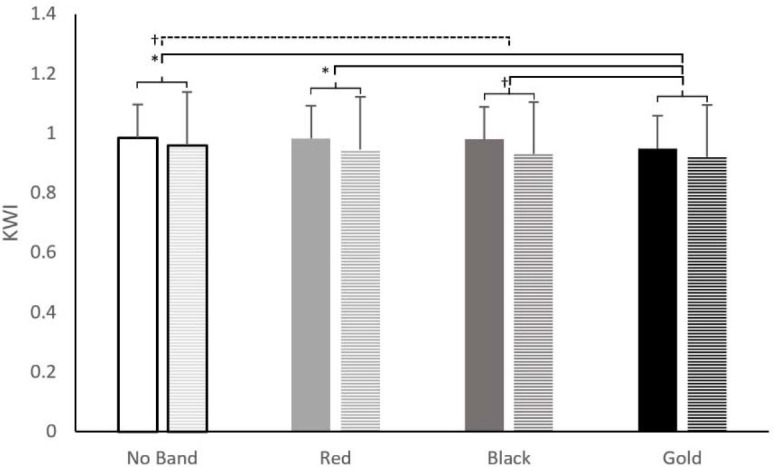
Knee-Width-Index (KWI) during a barbell back squat for different band conditions (No Band, Red, Black, Gold) in males (solid-coloured bars) and females (checkered bars). † p ˂ 0.05, * p ˂ 0.01

A repeated measures ANOVA revealed a significant main effect for the band on the KWI (F (2.37,99.53) = 21.91, *p* < 0.01, pη^2^ = 0.34) with the gold band showing a significantly smaller KWI compared to no band (↑4%, *p* < 0.01), red band (↑3%, *p* < 0.01) and black band (↑2%, *p* < 0.01) condition. Additionally, the black band had a KWI lower by 2% compared to no band condition (*p* < 0.01). The results also revealed a significant interaction effect for band × sex (F (2.37,99.53) = 3.02, *p* = 0.04, pη^2^= 0.07; Figure 6) on the KWI with males showing a significantly higher KWI compared to females in their respective bands. No other interaction effect was found for band × phase (F (2.37,99.53) = 0.26, *p* = 0.81), band × sex × phase (F (2.37,99.53) = 0.19, *p* = 0.83). The results for the maximum knee flexion showed that bands did not significantly affect maximum squat depth (ꭓ^2^(3) = 6.7, *p* = 0.08). The maximum knee flexion was not different between males and females for all conditions (no band, *p* = 0.79; red, *p* = 0.29, black; *p* = 0.39; gold, *p* = 0.95)

### Strength

For the 5RM squat relative to body mass, male participants (1.18 ± 0.16) demonstrated 28% greater strength compared to females (0.92 ± 0.26; t (44) = 3.98, *p* < 0.01, d = 1.15). Pearson’s two-tailed correlation analysis showed no correlation between strength and the KWI regardless of the squatting phase for all conditions (*p* = 0.06). When controlled for sex, however, females demonstrated a positive correlation between the KWI and strength during the ascending phases for red (r(9) = 0.61, *p* = 0.04) and gold (r(9) = 0.65, *p* = 0.03) bands; and during the descending phase for all conditions (no band: r(9) = 0.61, *p* = 0.04, red: r(9) = 0.67, *p* = 0.02, black: r(9) = 0.65, *p* = 0.03, gold: r(9) = 0.7, *p* = 0.02). However, male participants did not show a correlation between relative strength and the KWI for all bands during both ascending and descending phases (*p* = 0.16). A significant positive correlation was found between strength of both sexes and ES activity during the descending (no band: r(21) = 0.6, *p* < 0.01, red: r(21) = 0.67, *p* < 0.01, black: r(21) = 0.59, *p* < 0.04, and gold: r(21) = 0.59, *p* < 0.01), and ascending (no band: r(21) = 0.56, *p* < 0.01, red: r(21) = 0.61, *p* < 0.01, black: r(21) = 0.58, *p* < 0.01, and gold: r(21) = 0.52, *p* = 0.01) phases. The BF showed a positive correlation with strength during the ascending phase for no band (r(21) = 0.45, *p* = 0.03) and the black band (r(21) = 0.44, *p* = 0.04) for both males and females. When controlled for sex a significant positive correlation was shown between females’ strength and ES activity during the ascending phase of the squat for no band (r(9) = 0.7, *p* = 0.02), red band (r(9) = 0.62, *p* = 0.04), and black band (r(9) = 0.7, *p* = 0.02); but not for males. No further correlation was observed between strength and EMG activity (*p* = 0.21).

## Discussion

Overall, this study illustrated that increased stiffness of the resistance band during the BBS reduced the amount of the KWI (i.e., greater MKD) deviating the knee from the neutral alignment towards the midline of the body. Concomitantly, as stiffness of the resistance band increased, the amount of VL muscle activation increased during the ascending phase of the BBS. GMe muscle activity was greater during the ascending phase of the BBS regardless of the usage of resistance bands. Sex differences were notable in the form of muscle activity and MKD. More specifically, the results of this study highlight kinematic, relative strength and lower extremity muscle activation differences between males and females during a BBS while using different resistance bands. In general, males showed less MKD (i.e., greater KWI) compared to females in each testing condition (no band, red band, black band, and gold band) during the BBS.

Our results are in agreement with previous studies that reported greater MKD in females compared to males during various forms of squats (Graci et al., 2012; Wallace et al., 2008). In contrast, one study reported an increase in MKD in male participants compared to females during a BBS when using stronger resistance bands (Reece et al., 2020). MKD difference during the BBS between sexes could be partially explained by the difference in relative BBS strength and/or anatomical predisposition (e.g., quadriceps femoris muscle angle [Q-angle]) of the lower extremities. The Q-angle is another method of measuring the frontal knee alignment as the acute angle created from the line connecting the anterior superior iliac spine to the middle of the patella and from the middle of the patella to the tibial tubercle is considered (Hungerford and Barry, 1979). Females, in general, have a greater Q-angle (i.e., smaller KWI) compared to males, which could put them at a disadvantage for controlling MKD (Herrington and Nester, 2004; Livingston, 1998). Furthermore, it has been shown that people with higher Q-angles demonstrate greater MKD during squat movements (Stiffler et al., 2015). The difference in the Q-angle between males and females would increase when performing weight-bearing activities such as the BBS (Carreiro, 2009). Studies have shown that the value of the Q-angle is negatively linked with quadriceps torque production (Binder et al., 2001; Saç and Taşmektepligil, 2018), which is a key factor in controlling frontal knee alignment when squatting (Claiborne et al., 2006). This could partially explain why stronger females demonstrated less MKD when using the red and gold resistance bands during all phases of a BBS. Additionally, females demonstrated a positive correlation between VM and VL muscle activity with MKD during the ascending phase when using the black resistance band. As the black resistance band pulls the knees towards each other, females utilize more of their quadriceps muscles to maintain neutral knee alignment. It seems, however, that the gold resistance band exerted too much force on the knee in such a way that females were not able to prevent MKD and simultaneously recruit the quadriceps muscles to counter. Ideally participants should strive to maintain optimum frontal knee alignment as MKD has been linked to knee injuries such as patellofemoral disorder (Foss et al., 2014).

Other notable sex differences during the BBS were the variation in muscle activity of the lower extremity muscles when using different resistance bands. Stronger resistance bands (i.e., black and gold) showed greater VL activation in males compared to females during the BBS. As resistance bands got stronger, they induced greater amounts of MKD along with a higher amount of VL muscle activity. It appears that males recruit a greater amount of VL activity to overcome MKD that the stronger resistance bands would cause. This notion was tested in one study where individuals with greater MKD displayed VL muscle activation (Dinis et al., 2021) without the use of training resistance bands. One reason for higher VL muscle recruitment in males could be attributed to the relative strength differences compared to females as males showed greater relative strength. Additionally, relative BBS strength was correlated with ES muscle activity in both males and females regardless of the resistance band being used. This is to be expected as heavier loads lifted in the BBS may require increased muscle activity of the ES to resist trunk flexion (i.e., stabilization) (Aspe and Swinton, 2014). However, when accounted for resistance band strength, females showed a strong correlation between ES muscle activity and relative squat strength during no band, red band, and black band conditions while ascending. It appears that the gold band distracted females from resisting trunk flexion to focus more on resisting MKD. Other sex related differences in terms of muscle activation were the negative correlation of BF activity with MKD when using the red resistance band during the descend phase of the BBS. Only one study showed differences in BF activity between males and females when performing a BBS, however, no resistance bands were used (Mehls et al., 2020). It remains to be elucidated why the red resistance band did induce such an effect on BF muscle activity in females.

Regardless of sex, GMe showed higher muscle activity when using the gold resistance band compared to all other conditions. This result is in agreement with two previous studies (Foley et al., 2017; Spracklin et al., 2017). However, two other studies did not find any difference in GMe activity (Forman et al., 2021; Reece et al., 2020). One main discrepancy would be the different resistance bands used in this study and a study of Reece and colleagues (2020). While the highest resistance band (gold) used in this study produced a maximum resistance of 6.44 kg at 100% elongation, the lowest resistance band in the study of Reece et al. (2020) produced 9.07 kg. Another important difference to point out is the type of the squat used in this study and the study by Forman et al. (2017). The squat used in Forman et al.’s (2018) study was an overhead squat which has shown to produce less activation of lower extremity muscles compared to the BBS. This is likely because during the BBS the individual is able to generate higher peak force, resulting in greater muscle activity. The increase in GMe activation for the gold resistance band can be justified by the amount of MKD induced. The stronger resistance bands (i.e., black and gold) induced higher amounts of MKD compared to no band condition during the BBS. In addition, the gold resistance band induced greater MKD compared to all other bands as well making it more challenging to maintain proper knee alignment. Similar results were derived from other studies showing that high-tension resistance bands resulted in greater knee valgus (Foley et al., 2017; Forman et al., 2021; Gooyers et al., 2012; Reece et al., 2020). To overcome MKD induced by the gold band the GMe would need to increase muscle activity to maintain its neutral knee alignment. The maintenance of neutral knee alignment would prevent the power absorption shift from the hip to the ankle, which would result in decreased power production (Pantano et al., 2005). The GMe muscle activation was higher during the ascending phase which is supported by previous research (Foley et al., 2017). Previous studies have highlighted the contribution of hip musculature strength in the frontal plane knee motion during squats and it was shown to be a significant predictor of frontal knee motion (Claiborne et al., 2006). It was shown that greater hip abduction strength would prevent MKD during squat movement (Claiborne et al., 2006). In addition to squats, the use of resistance bands at the distal portion of the thigh has shown to increase GMe activation at landing, having the potential benefit of recruiting muscles when omitting the resistance band during a functional task (Dai et al., 2014). The increased activation of the GMe with the use of a resistance band during BBS could promote neutral knee alignment when not using the band (Hoogenboom et al., 2018) and can be considered an effective exercise for increasing GMe activity.

In contrast to increased GMe muscle activation, participants showed decreased VM muscle activity during the BBS when using black and gold resistance bands. Similarly, another study showed a decrease in VM muscle activity, however, the reduction in VM muscle activation was not statistically different (Reece et al., 2020). Other studies have shown that with an increase in the Q-angle, which is similar to the alignment of the knee when experiencing MKD, individuals exhibited less knee extensor torque (Binder et al., 2001; Saç and Taşmektepligil, 2018). When taking into consideration the amount of MKD induced with the same bands compared to no band condition, it is likely that due to change in knee alignment, it would put the VM at a disadvantage by increasing the moment arm of the muscle causing a reduction in VM activation and relying on other muscles such as the GMe as it also assists in the hip extension moment.

There are some limitations to consider when generalising the results of this study. One factor that may have influenced the findings of the present study is the inherent squat quality differences between our male and female participants prior to using the elastic bands. It is possible that males and females exhibit different lower limb kinematics and muscle activity patterns while performing the BBS, leading to unique responses when adding an elastic band. Therefore, future research studies should account for this possibility and attempt to control and match BBS characteristics between sexes. Additionally, the EMG data were collected unilaterally when performing the BBS from the dominant leg. Although a previous study by Froman et al. (2018) did not find any difference in muscle activation between the two legs while performing a BBS, it can be noted that the non-dominant leg may be more prone to displacement and therefore alter muscle activation compared to the dominant leg. Thus, further experimentation is required to clarify the differences in bilateral muscle activation during a BBS when considering MKD. Another potential limitation was that trunk kinematics while performing the BBS was not measured. Males and females from this study might have exhibited unique trunk inclination prior to using the bands, and this might have influenced our findings. Lastly, female and male adaptation to resistance bands during the BBS could be associated with changes in MKD. It is possible that female kinematics compensate differently from males, when using resistance bands. This concept warrants future research as well.

Using greater stiffness resistance bands around the distal thighs while performing the BBS can cause deviation in knee alignment by increasing MKD. Furthermore, it should be noted that females tend to exhibit more MKD compared to males when using the same resistance band during the BBS. As a result, females may be at a biomechanical disadvantage when using resistance bands compared to males while performing the BBS hindering them from optimal performance. By increasing the relative BBS, females may improve their frontal knee alignment when using resistance bands. Taking into account the muscle activation, elastic bands may improve gluteal muscle activation, and consequently improve MKD, but minimal longitudinal work has been performed to date (specifically on athletes who are already demonstrating MKD). It is therefore uncertain whether elastic bands provide enough benefit to warrant their use. What does seem to be clear at the moment is that, if one chooses to use elastic bands while squatting, lighter bands appear to be superior. From previous work, they provide the same possible benefits as stronger bands, but with less risk of enhancing MKD.
